# Chikungunya virus vector competency of Brazilian and Florida mosquito vectors

**DOI:** 10.1371/journal.pntd.0006521

**Published:** 2018-06-07

**Authors:** Nildimar Alves Honório, Keenan Wiggins, Daniel Cardoso Portela Câmara, Bradley Eastmond, Barry W. Alto

**Affiliations:** 1 Laboratório de Mosquitos Transmissores de Hematozoários, Instituto Oswaldo Cruz, Fundação Oswaldo Cruz, Rio de Janeiro, Brasil; 2 Núcleo Operacional Sentinela de Mosquitos Vetores-Nosmove/Fiocruz, Fundação Oswaldo Cruz, Rio de Janeiro, Brasil; 3 University of Florida, IFAS, Department of Entomology and Nematology, Florida Medical Entomology Laboratory, Vero Beach, FL, United States of America; Faculty of Science, Mahidol University, THAILAND

## Abstract

Chikungunya virus is a vector-borne alphavirus transmitted by the bites of infected female *Ae*. *aegypti* and *Ae*. *albopictus*. In Brazil between 2014 and 2016 almost 320 thousand autochthonous human cases were reported and in Florida numerous imported CHIKV viremic cases (> 3,800) demonstrate the potential high risk to establishment of local transmission. In the present study, we carried out a series of experiments to determine the viral dissemination and transmission rates of different Brazilian and Florida populations of *Ae*. *aegypti* and *Ae*. *albopictus* at 2, 5, and 13 days post-infection for the emergent Asian genotype of CHIKV. Our results show that all tested populations of *Ae*. *aegypti* and *Ae*. *albopictus* have a high proportion (> 0.80) of individuals with disseminated infection as early as 2 days-post exposure. We found no significant treatment effects of mosquito population origin effects on viral dissemination rates. Transmission rates had a heterogeneous pattern, with US *Ae*. *aegypti* and Brazilian *Ae*. *albopictus* having the highest proportion of individuals with successful infection (respectively 0.50 and 0.82 as early as 2 days-post infection). Model results found significant effects of population origin, population origin x species, population origin x days post-infection and population origin x species x days post infection.

## Author summary

Chikungunya is considered a serious mosquito-borne disease in many tropical and subtropical countries throughout the world. It is already an epidemic disease in Brazil and poses as a potential risk in Florida. It is mainly transmitted by mosquitoes *Aedes aegypti* and *Aedes albopictus*. These mosquito species are common and abundant throughout much of the year in Brazil and Florida. In this study, we determined two components of vector competence from Brazilian and Florida populations of both mosquitoes to the emergent Asian genotype of chikungunya virus: viral dissemination and transmission rates. Both *Aedes* populations exhibited a high proportion of disseminated infection as early as two days after ingestion of chikungunya virus infected blood. Transmission efficiency was higher in *Ae*. *aegypti* from Florida and *Ae*. *albopictus* from Brazil. Our findings suggest that mosquito-virus interactions of both *Ae*. *aegypti* and *Ae*. *albopictus* may vary by geographic population, which may impact public health measures and should be considered during outbreaks of this arboviral disease.

## Introduction

Chikungunya fever is a vector-borne viral disease that originated in Africa and is caused by a virus (CHIKV; family *Togaviridae*, genus *Alphavirus*) transmitted by the bites of infected female *Aedes* mosquitoes, mainly *Ae*. *aegypti* and *Ae*. *albopictus* [1]. There are three genotypes of CHIKV, which apparently evolved independently in distinct geographic regions: Asian, West African, and East/Central/South African (ECSA) [2]. CHIKV is widespread worldwide and poses as a major public health problem in tropical and subtropical regions [3–6]. In the Americas, autochthonous transmission of CHIKV was first detected in St. Martin Island in October 2013 and quickly spread throughout the Americas in the following months [7–9]. The initial spread of autochthonous cases in the Americas was due to the Asian genotype, but the ECSA genotype was also detected circulating in Brazil in 2014 [10]. To date, local transmission of CHIKV has been documented in over 43 countries with more than 1,000,000 confirmed cases, where Brazil reported 314,834 until the 15^th^ epidemiological week of 2017 [11–12].

*Aedes aegypti* and *Ae*. *albopictus* are the main vectors of CHIKV, and both are highly invasive species and closely associated with the human peridomestic environment [13, 14, 6]. *Aedes aegypti* is highly anthropophilic and exhibits endophilic behavior and is mostly associated with high human density. In contrast, *Ae*. *albopictus* shows an eclectic feeding behavior, preferentially feeding and resting in the peridomicile and is more common in vegetated and urban/urban forest transition habitats, especially where it is sympatric with *Ae*. *aegypti* [15–19]. In Africa, CHIKV is maintained via an enzootic cycle involving several species of arboreal mosquitoes, including *Ae*. *africanus* and *Ae*. *furcifer*, and non-human primates [20]. Epidemic transmission is maintained mainly by *Ae*. *aegypti* in urban environments, but a single-base mutation in a strain of the ECSA genotype during the outbreak in La Réunion Island enhanced vector competence of *Ae*. *albopictus* [21, 22]. A second mutation is associated with enhanced vector competence of *Ae*. *albopictus* during an outbreak in Kerala, India [23]. In fact, the acquisition of second-step *Ae*. *albopictus*-adaptive mutations by CHIKV strains might indicate even more efficient transmission by this invasive vector [24].

Vector competence studies are important to determine the potential of resident mosquito populations to transmit CHIKV. Vector competence is a phenotypic parameter that describes the ability of the vector to become infected, replicate and transmit a pathogen [25, 26]. Moreover, vector competence depends on vector and viral genetic characteristics [27] and environmental factors such as ambient temperature and diurnal temperature range [28–32]. It has been shown that vector competence of *Ae*. *aegypti* and *Ae*. *albopictus* for CHIKV is a complex interaction dependent on vector population, virus strain and temperature [33, 34]. The vector competence of *Ae*. *aegypti* for dengue virus (DENV) has been shown to have high variability and heterogeneity whether it is analyzed at city [35], country [36] or continental level [37].

Previous studies of CHIKV have characterized variation in vector competence among CHIKV genotypes, extrinsic incubation temperature, and geographic populations of *Ae*. *aegypti* and *Ae*. *albopictus*, and species-specific differences. In Florida, *Ae*. *aegypti* and *Ae*. *albopictus* were highly susceptible to infection and viral dissemination to ECSA and Asian genotypes of CHIKV, with some variation between strains [38, 39]. Pesko et al. (2009) [40] evaluated vector competence of *Ae*. *aegypti* and *Ae*. *albopictus* from Florida for infection with a La Réunion island ECSA isolate of CHIKV. Although both species were susceptible to high CHIKV doses, *Ae albopictus* was more susceptible to infection than *Ae*. *aegypti*. Richards et al. (2010) [33] assessed the effect of extrinsic incubation temperature on vector competence of Florida mosquitoes for CHIKV isolates from La Réunion and found highest infection, dissemination, and transmission rates in *Ae*. *albopictus* than in *Ae*. *aegypti* and *Culex quinquefasciatus*, but no effect on the extrinsic incubation period. Vega-Rúa et al. (2014) [31] working with three CHIKV genotypes and 35 populations of *Ae*. *aegypti* and *Ae*. *albopictus* mosquitoes from 10 American countries showed that all *Aedes* populations tested were susceptible to CHIKV infection by all three genotypes. However, CHIKV transmission was heterogeneous in American *Ae*. *aegypti* and *Ae*. *albopictus* populations, ranging from 11.1% to 96.7%. In this study, the *Aedes* populations from Rio de Janeiro showed high transmission rates, and *Ae*. *albopictus* from Florida were more competent vectors than *Ae*. *aegypti*.

Although *Ae*. *aegypti* is considered the primary epidemic vector of CHIKV and *Ae*. *albopictus* a potential vector in some areas [2, 21, 31], heterogeneous vector competence of both species may alter risk of disease transmission, as evidenced by the participation of *Ae*. *albopictus* in the outbreak in La Réunion Island [21]. Studies comparing vector competence in American populations of both species are necessary in a scenario where travel and global trade in endemic regions have increased the risk for spread of CHIKV, as evidenced by its introduction in the Americas [41]. Also, there is a real risk for the introduction of CHIKV strains with adaptive mutations to enhance vector competence of *Ae*. *albopictus*, an invasive species which is widespread in the Americas [24. With the aim to shed light on the causes and consequences of geographical variations in the transmission of arboviruses of public health concern, we carried out an experiment to determine the dissemination and transmission rates of Brazilian and Florida populations of *Ae*. *aegypti* and *Ae*. *albopictus* for the emergent Asian genotype of CHIKV.

## Materials and methods

### Ethics statement

Chikungunya virus (Asian lineage, GenBank accession: KJ451624) used was isolated from the serum of an infected human in the British Virgin Islands in 2013 by other investigators. Subsequently, this isolate was archived with the Centers for Disease Control and Prevention. We requested an isolate of this virus for use in this study and so the sample was already present in an already-existing collection (Centers for Disease Control and Prevention, Arboviral Diseases Branch). The virus sample was anonymized and Institutional Review Board approval was not needed for receipt and use of the sample in this study. No entomological gathering was done on private land or in private residence for this study.

### Mosquito collections and rearing

**Fig 1:**
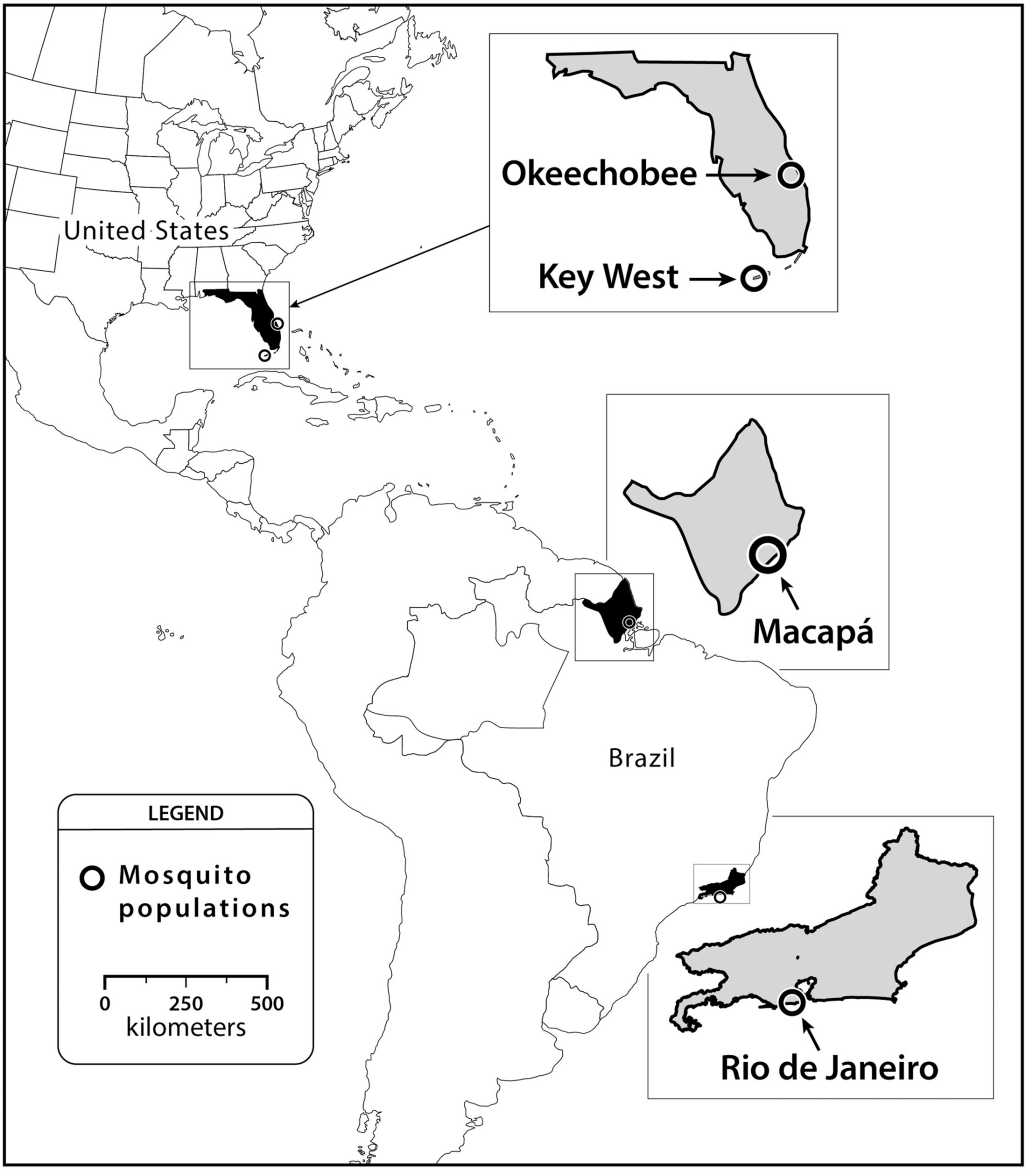
Location of *Ae*. *aegypti* and *Ae*. *albopictus* collected from allopatric and sympatric populations in Brazil and United States.

The *Ae*. *aegypti* and *Ae*. *albopictus* populations used in this experiment were collected in Rio de Janeiro (RJ) and Macapá (MC)—Brazil, Key West (KW) and Okeechobee (OK), Florida—United States (Fig 1, Table 1). All gathering of entomological samples were done on public land. We chose collection sites based on allopatric *Ae*. *aegypti* to *Ae*. *albopictus* (MC and KW) and sympatric populations (RJ and OK). Some of these areas report local transmission of chikungunya cases (RJ and MC) while others are located near regions in Florida where local transmission has occurred (Miami-Dade, Palm Beach, St. Lucie, and Broward Counties) (KW and OK) [12, 42]. In Brazil, eggs of both species were obtained from oviposition traps during a routine entomological survey. *Aedes albopictus* from a sympatric population (RJ) were obtained at the Oswaldo Cruz Foundation campus, in the Manguinhos neighborhood in March 2015 from 50 oviposition traps using methods described elsewhere [16]. *Aedes aegypti* eggs from an allopatric population (MC) were collected with oviposition traps by personnel from the Amapá State Health Secretary in May 2015. In United States, eggs of allopatric *Ae*. *aegypti* (KW) were collected in March 2015 with oviposition traps by personnel of Florida Keys Mosquito Control District. Immatures of sympatric *Ae*. *albopictus* (OK) were collected from tires in October 2015.

**Table 1:** Mosquito populations used in this study by country of collection from Brazil and the United States.

Country	Location	coordinates	Species	Climate	Strain	Generation tested
Brazil	Macapá, Amapá	0°02'N 51°04' W	*Ae*. *aegypti*	Tropical Wet	Allopatric	F3
Brazil	Manguinhos, Rio de Janeiro	22°52’S 43°14’W	*Ae*. *albopictus*	Tropical Wet and Dry	Sympatric	F3
United States	Key West, Florida	24°33'N 81°46'W	*Ae*. *aegypti*	Tropical savanna	Allopatric	F3
United States	Okeechobee, Florida	27°14'N 80°50'W	*Ae*. *albopictus*	Humid subtropical	Sympatric	F2

Field-collected mosquitoes (eggs or larvae) were reared in pans containing 1 L of tap water (100 larvae per pan) to adulthood on a diet with 0.6 g of equal amounts of brewer’s yeast and lactalbumin. Mosquitoes were held in a climate controlled room at 26–28°C and a photoperiod of 14:10 hours light:dark. Upon pupation, pupae were collected daily and placed in vials with a cotton seal until eclosion after which adult mosquitoes were identified to species. Adults were transferred to 0.3m^3^ cages and provided with 10% sucrose solution and water from cotton wicks and allowed to feed on bovine blood once per week using an artificial feeding system with hog intestine membranes. Females and males were held together for eleven days after which females were transferred to cylindrical cages (ht. by dia., 10 cm by 10 cm, 50 females/cage) with mesh screening one day before being fed CHIKV infected blood. The F_2_ (Okeechobee) and F_3_ (Rio de Janeiro, Macapá and Key West) generations progeny of field-collected *Ae*. *aegypti* and *Ae*. *albopictus* were used for the CHIKV infection study.

### Virus and mosquito oral infection

The strain of CHIKV (Asian lineage, GenBank accession: KJ451624) used was isolated from the serum of an infected human in the British Virgin Islands in 2013. The Centers for Disease Control and Prevention was the source of the virus strain used in this study. The CHIKV isolate was passaged twice in culture using African green monkey (Vero) cells and viral titer was determined in 6-well plates seeded with Vero cells (American Type Culture Collection, ATCC) by plaque assay using a modified procedure by Kaur et al. (2016) [43].

For preparation of the virus suspension, monolayers of Vero cells were inoculated with dilute stock CHIKV at a multiplicity of infection of 0.1 followed by a one-hour incubation at 37°C and 5% carbon dioxide atmosphere. The American Type Culture Collection was the source of Vero cells used in this study. After the inoculation procedure, each flask received 24 ml media (M199 medium supplemented with 10% fetal bovine serum, penicillin/streptomycin and mycostatin) and was left to incubate for an additional 47-hours. Adult females aged 10–11 days were offered CHIKV infected defibrinated bovine blood (Hemostat, Dixon, CA) using an artificial feeding system with hog intestine membranes (Hemotek, Lancashire, United Kingdom). Samples of blood were taken of the virus-blood suspension at the time of feeding to determine the concentration of CHIKV ingested by the adult mosquitoes. Blood meal titers ranged from log_10_ 7.3 to 8.3 plaque forming unit equivalents (pfue)/mL. Fully engorged females were held in cylindrical cages along with an oviposition substrate and maintained at a 14:10 hour light:dark photoperiod and 28°C.

Virus transmission potential using saliva assays was determined at 2, 5, and 13 days after feeding on infected blood. Mosquitoes were deprived of sucrose for 1-day and then individually transferred to plastic tubes fitted with a removable screen lid (37-mL 8 by 3 cm). Honey was dyed with blue food coloring (McCormick) and impregnated on filter paper (1 cm diameter) and fastened to the inside lid of the tube. Mosquitoes that fed on the honey deposited saliva and the blue food coloring was visualized in the crop with aid of an incandescent flashlight. Mosquitoes were examined for blue in the crop after 24 and 48-hours during the transmission assay. Only mosquitoes that fed on honey were used to assess transmission potential. Additionally, saliva was collected from another subset of mosquitoes in capillary tubes with immersion oil as described previously [44,32, 39]. Mosquitoes were stored at -80°C after the transmission assay and later dissected to test the legs and saliva for the presence of CHIKV RNA by qRT-PCR [32]. The sequence of primers targeting a nonstructural polyprotein gene was as follows: forward, 5'-GTACGGAAGGTAAACTGGTATGG-3': reverse, 5'-TCCACCTCCCACTCCTTAAT-3'. The probe sequence was: 5'-/56-FAM/TGCAGAACCCACCGAAAGGAAACT/3BHQ_1/-3' (Integrated DNA Technologies, Coralville, IA). Detection of CHIKV RNA in the legs of a mosquito is considered a proof that the virus infection has disseminated from the midgut, and we use the number of mosquitoes with a disseminated infection over the number of mosquitoes fully engorged on a viraemic blood-meal, as the virus dissemination rate. Detection of CHIKV RNA in mosquito saliva is considered a proof that the mosquito can transmit virus when feeding, and we use the proportion of mosquitoes with virus in saliva among all mosquitoes with a disseminated infection as our expression of transmission rate.

For each mosquito, legs were triturated in 1.0 mL of media (GIBCO Media 199). Saliva from mosquitoes was combined with 300 μL of media. RNA isolation on a 140 μL sample of mosquito legs and saliva homogenate was achieved using the QIAamp viral RNA mini kit (Qiagen, Valencia, CA) and eluted in 50 μL of buffer according to the manufacturer’s protocol. Viral RNA was detected using the Superscript III One-Step qRT-PCR with Platinum Taq kit by Invitrogen (Invitrogen, Carlsbad, CA) using methods described elsewhere [32, 39]. Quantitative RT-PCR was performed with the CFX96 Real-Time PCR Detection System (Bio-Rad Laboratories, Hercules, CA) with the following program: 50°C for 30 minutes, 94°C for 2 minutes, 39 cycles at 94°C for 10 seconds and 60°C for 1 minute, and 50°C for 30 seconds. The expression of viral titer in mosquito-derived samples used a standard curve method comparing cDNA synthesis for a range of serial dilutions of CHIKV in parallel with plaque assays of the same dilutions of virus, expressed as plaque forming unit equivalents (pfue)/ml [45].

### Statistical analyses

We were interested in analyzing the relationship between the presence or absence of CHIKV in the legs and saliva (dependent variables) and the following independent variables: mosquito species (*Ae*. *aegypti* and *Ae*. *albopictus*), population origin (Brazil and USA), days post-infection (dpi, 2, 5 and 13), and a three-way interaction of species by population origin by days post-infection. Exploratory analyses were done using chi-square tests to verify possible relationships between both dependent variables (presence or absence of CHIKV in the legs and saliva) and each of the independent variables. We modeled this relationship using two separate binomial generalized linear models: one focused on the viral dissemination to the legs, and the other focused on the viral infection of saliva. To account for numerical problems in the viral dissemination binomial model, we used a Firth's Bias-Reduced Logistic Model [46]. We also analyzed the relationship between the viral titer of legs and saliva and the aforementioned main effects using a Gaussian generalized linear model. All analyses were done using R [47] and RStudio [48], with the libraries ggplot2 [49], logistf [46] and lsmeans [50].

### Accession numbers

Chikungunya virus (Asian lineage, GenBank accession: KJ451624, repository: Centers for Disease Control and Prevention).

## Results

### Chikungunya virus disseminated infection by species, population origin and days post-infection

Chikungunya virus dissemination rates were measured by the proportion of mosquitoes that had infected legs from the total that fully engorged on infected blood. A total of 358 *Aedes* mosquitos were tested for disseminated infection (172 *Ae*. *aegypti* and 186 *Ae*. *albopictus*). Overall, our results showed the proportion of individuals of both species with disseminated infection significantly increased with each of the days post-infection analyzed (2-dpi, 0.847 ± 0.034; 5-dpi, 0.977 ± 0.013; and 13 dpi, 0.984 ± 0.011) (χ^2^ = 24.35, df = 2, p<0.0001). *Aedes aegypti* had higher dissemination rates than *Ae*. *albopictus* (mean ± SE, 0.960 ± 0.014 and 0.919 ± 0.020, respectively), although not significant (χ^2^ = 2.09, df = 1, p = 0.148). Both US and Brazilian populations of *Ae*. *aegypti* (0.976 ± 0.016 and 0.946 ± 0.023, respectively) had higher dissemination rates when compared to *Ae*. *albopictus* (0.915 ± 0.028 and 0.922 ± 0.028, respectively), but this difference was also not significant (χ^2^ = 0.03, df = 1, p = 0.857).

**Fig 2:**
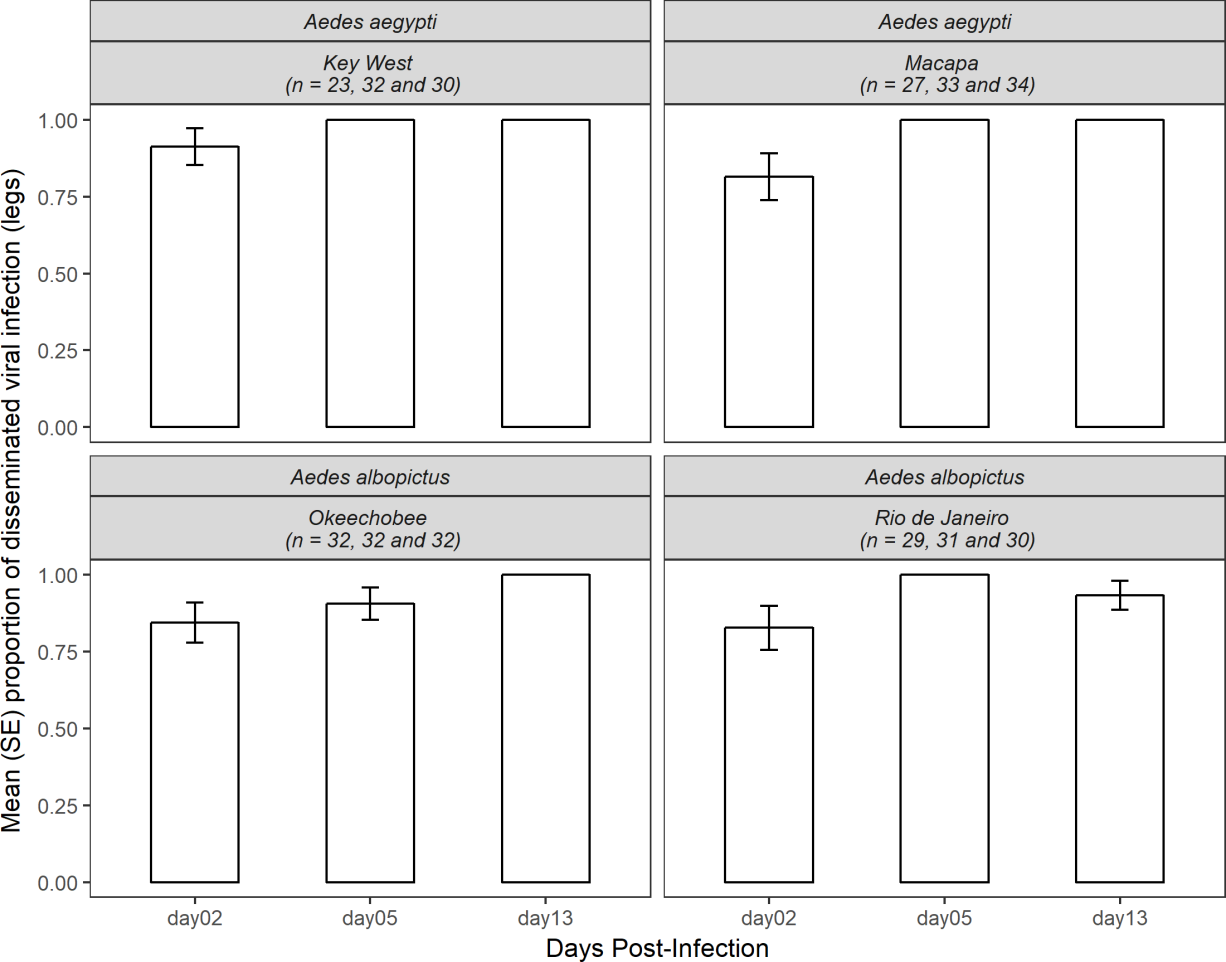
Mean (SE) dissemination rate of CHIKV in *Ae*. *aegypti* and *Ae*. *albopictus* of Key West, Macapá, Okeechobee and Rio de Janeiro populations at 2, 5 and 13 days post-infection. Columns lacking error bars display 100% infection.

When analyzing the dissemination rates per species, population origin and days post-infection interaction, *Ae*. *aegypti* reached 100% of individuals at the 5^th^ and 13^th^ days, but the US population had higher dissemination rates at the 2^nd^ day when compared to the Brazilian population (0.913 ± 0.06 and 0.814 ± 0.07, respectively) (Fig 2). These differences, however, were not significant (χ^2^ = 0.07, df = 2, p = 0.961). For *Ae*. *albopictus*, both US and Brazilian populations had similar dissemination rates at the 2^nd^ day (0.843 ± 0.065 and 0.827 ± 0.071). At the 5^th^ day, the US population had a lower dissemination rate when compared to the Brazilian population (0.906 ± 0.052 and 1.0, respectively). At the 13^th^ day, the *A*. *albopictus* US population had a higher dissemination rate (1.0) than the Brazilian population (0.933 ± 0.046). The dissemination rate did not significantly differ between population origins (χ^2^ = 0.36, df = 2, p = 0.834) (Fig 2).

The three-way interaction Firth's bias-reduced logistic model results show that none of the main effects or the interactions were significant for disseminated infection rates (Table 2).

**Table 2:** Estimated effects of the population origin, species and days post-infection on dissemination rates and viral titer of chikungunya virus in *Ae*. *aegypti* and *Ae*. *albopictus*. Bold entries indicate statistical significance (p < 0.05).

Response	Effect	Estimate	Standard Error	95% CI	
				Lower Bound	Upper Bound
**Disseminated infection**	**(Intercept)**	**2.499**	**0.73**	**1.465**	**4.835**
	Origin:US	0.646	1.287	-1.763	5.149
	Species: *Ae*. *albopictus*	0.069	1.03	-2.252	2.306
	Days:05	2.639	2.586	0.204	10.686
	Days:13	2.659	2.578	0.011	10.7
	Origin:US x Species: *Ae*. *albopictus*	-0.551	1.644	-4.67	2.552
	Origin:US x Days:05	-0.666	3.743	-9.026	7.305
	Origin:US x Days:13	-0.729	3.75	-8.72	7.631
	Species: *Ae*. *albopictus* x Days:05	-0.111	3.67	-8.47	7.874
	Species: *Ae*. *albopictus* x Days:13	-1.84	2.873	-9.92	2.482
	Origin: US x Species: *Ae*. *albopictus* x Days:05	-1.413	4.7	-9.85	7.025
	Origin: US x Species: *Ae*. *albopictus* x Days:13	2.365	4.732	-6.072	10.803
**Viral titer in the legs**	**(Intercept)**	**2.181**	**0.287**	**1.619**	**2.743**
	Origin:US	0.468	0.423	-0.361	1.297
	Species: *Ae*. *albopictus*	0.378	0.399	-0.404	1.159
	**Days:05**	**1.430**	**0.387**	**0.672**	**2.188**
	**Days:13**	**1.938**	**0.384**	**1.185**	**2.691**
	**Origin:US x Species: *Ae*. *albopictus***	**-1.261**	**0.570**	**-2.378**	**-0.144**
	Origin:US x Days:05	0.210	0.562	-0.891	1.311
	Origin:US x Days:13	-0.456	0.564	-1.562	0.649
	Species: *Ae*. *albopictus* x Days:05	-0.017	0.546	-1.087	1.052
	Species: *Ae*. *albopictus* x Days:13	-0.774	0.546	-1.845	0.296
	Origin: US x Species: *Ae*. *albopictus* x Days:05	0.324	0.776	-1.197	1.846
	Origin: US x Species: *Ae*. *albopictus* x Days:13	1.203	0.780	-0.324	2.731

When analyzing the viral titers in the mosquito legs, Gaussian model results show that days post-infection had a significant positive effect, and the interaction of species and population origin had a significant negative effect (Table 2). Overall, both populations of *Ae*. *aegypti* had lower levels of viral titer (expressed in log_10_ pfue/mL) in their legs at 2^nd^ day post-infection, which increased and peaked at the 5^th^ and 13^th^ days (US; 2^nd^ day = 2.884 ± 0.453, 5^th^ day = 4.289 ± 0.179 and 13^th^ day = 4.131 ± 0.053; and Brazilian 2^nd^ day = 2.668 ± 0.411, 5^th^ day = 3.610 ± 0.277 and 13^th^ day = 4.119 ± 0.110). The same pattern was observed for *Ae*. *albopictus* for both US (2^nd^ day = 2.060 ± 0.290, 5^th^ day = 4.074 ± 0.263 and 13^th^ day = 3.676 ± 0.244) and Brazilian populations (2^nd^ day = 3.086 ± 0.362, 5^th^ day = 3.971 ± 0.241 and 13^th^ day = 3.988 ± 0.183) (S1 Fig).

### Chikungunya virus saliva infection by species, population origin and days post-infection

**Fig 3:**
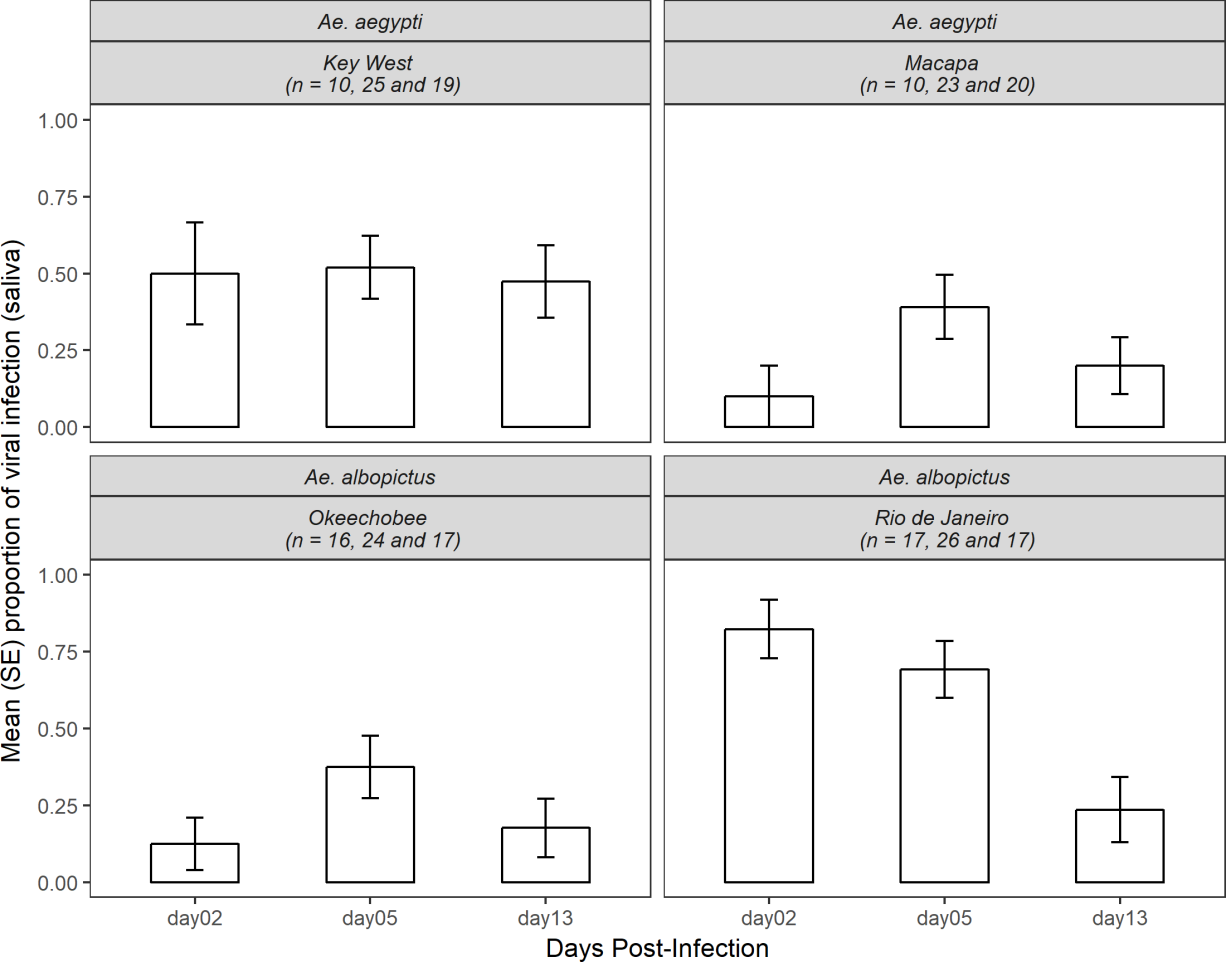
Mean (SE) proportion of CHIKV saliva infection in *Ae*. *aegypti* and *Ae*. *albopictus* of Key West, Macapá, Okeechobee and Rio de Janeiro populations at 2, 5 and 13 days post-infection.

Chikungunya virus infection rates were measured by the proportion of mosquitoes that had infected saliva from the total that presented viral dissemination. A total of 224 *Aedes* mosquitoes that had positive leg infections were tested for saliva infection (107 *Ae*. *aegypti* and 117 *Ae*. *albopictus*). Overall, we found a significant effect of days post-infection and infection rates when analyzing both species (χ^2^ = 8.88, df = 2, p<0.05) (Fig 3). The infection rates reached a peak at the 5^th^ day post-infection and decreased at the 13^th^ day (2-dpi, 0.415 ± 0.068; 5-dpi, 0.500 ± 0.050; and 13-dpi, 0.274 ± 0.053). We also found a significant relationship between infection rates per species and population origin (χ^2^ = 11.55, df = 1, p<0.0001); US *Ae*. *aegypti* had higher infection rates when compared to the Brazilian (*Ae*. *aegypti*, 0.5 ± 0.068; *Ae*. *albopictus*, 0.264 ± 0.061). For *Ae*. *albopictus*, the US population had lower infection rates when compared to Brazilian conspecifics (0.245 ± 0.057 and 0.6 ± 0.063, respectively). The analysis of infection rates per species, population origin and days post-infection for *Ae*. *aegypti* showed that the US population had similar rates in all days (2-dpi, 0.5 ± 0.166; 5-dpi, 0.52 ± 0.101; 13-dpi, 0.473 ± 0.117). The Brazilian population had a lower infection rate when compared with the US population at all day’s post-infection (0.1 ± 0.1, 0.391 ± 0.2 and 0.104 ± 0.091, respectively), although this difference was not significant (χ^2^ = 1.32, df = 2, p = 0.67). For *Ae*. *albopictus*, the US population had a lower infection rate at the 2^nd^ and 13^th^ days (0.125 ± 0.085 and 0.176 ± 0.095, respectively) and higher infection rates at the 5^th^ day (0.375 ± 0.1). The Brazilian population however had high infection rate at the 2^nd^ day (0.823 ± 0.095), decreasing at the 5^th^ day (0.692 ± 0.092) and finally decreasing further at the 13^th^day (0.235 ± 0.106). The Brazilian population had a higher infection rate at all day’s post-infection when compared to the US population, but this difference was not significant (Fig 2, χ^2^ = 3.05, df = 2, p = 0.238) (Fig 3).

The three-way interaction logistic model results showed a significant effect of population origin, and the interactions between population origin x species, population origin x days post-infection and the three-way interaction of population origin x species x days post infection were significant for saliva infection rates (Table 3).

**Table 3:** Estimated effects of the population origin, species and days post-infection on the saliva infection rates and viral titer of caribbean CHIKV in *Ae*. *aegypti* and *Ae*. *albopictus*. Bold entries indicate statistical significance (p < 0.05).

Response	Effect	Estimate	Standard Error	95% CI	
				Lower Bound	Upper Bound
**Saliva infection**	**(Intercept)**	**-2.197**	**1.054**	**-5.112**	**-0.526**
	**Origin:US**	**2.197**	**1.2292**	**0.058**	**5.3**
	Species: *Ae*. *albopictus*	3.738	1.2311	1.658	6.86
	Days:05	1.755	1.1373	-0.151	4.755
	Days:13	0.811	1.1931	-1.284	3.864
	**Origin:US x Species: *Ae*. *albopictus***	**-5.684**	**1.5771**	**-9.289**	**-2.851**
	Origin:US x Days:05	-1.675	1.3615	-4.935	0.776
	**Origin:US x Days:13**	**-0.916**	**1.4264**	**-4.253**	**1.704**
	Species: *Ae*. *albopictus* x Days:05	-2.485	1.3707	-5.771	-0.052
	Species: *Ae*. *albopictus* x Days:13	-3.53	1.468	-6.946	-0.873
	**Origin: US x Species: *Ae*. *albopictus* x Days:05**	**3.84**	**1.7856**	**0.534**	**7.745**
	**Origin: US x Species: *Ae*. *albopictus* x Days:13**	**4.041**	**1.9345**	**0.392**	**8.172**
**Viral titer in the saliva**	(Intercept)	0.066	0.264	-0.450	0.582
	**Origin:US**	**0.831**	**0.373**	**0.101**	**1.561**
	**Species: *Ae*. *albopictus***	**0.836**	**0.332**	**0.185**	**1.487**
	Days:05	0.603	0.316	-0.015	1.222
	Days:13	0.217	0.323	-0.415	0.850
	**Origin:US x Species: *Ae*. *albopictus***	**-1.386**	**0.479**	**-2.324**	**-0.447**
	Origin:US x Days:05	-0.677	0.444	-1.546	0.193
	Origin:US x Days:13	-0.444	0.458	-1.342	0.454
	Species: *Ae*. *albopictus* x Days:05	-0.464	0.409	-1.265	0.338
	Species: *Ae*. *albopictus* x Days:13	-0.811	0.431	-1.656	0.034
	Origin: US x Species: *Ae*. *albopictus* x Days:05	0.593	0.586	-0.554	1.741
	Origin: US x Species: *Ae*. *albopictus* x Days:13	0.850	0.618	-0.361	2.062

The Gaussian model to analyze the viral titer in the saliva of the tested mosquitoes did not detect significant main effects or interactions of the treatment factors (Table 3). The US population of *Ae*. *aegypti* had similar levels of viral titer in the saliva at all three time-points tested (respectively 1.794 ± 0.593, 1.516 ± 0.247 and 1.351 ± 0.171 pfue/mL), while the Brazilian population had a peak at the 5^th^ day and decreasing at the 13^th^ day (respectively 1.659 ± 0.376 and 1.300 ± 0.429 pfue/mL). The US population of *Ae*. *albopictus* had higher viral titer in their saliva at the 2^nd^ day, decreasing with each passing time point (2.290 ± 0.730, 1.036 ± 0.247 and 0.810 ± 0.228 pfue/mL). For the Brazilian population of this species, viral titer peaked at 5^th^ days, decreasing at the 13^th^ (respectively 1.095 ± 0.140, 1.501 ± 0.245 and 1.058 ± 0.458 pfue/mL) (S2 Fig).

## Discussion

This study tested the vector competence of two populations of *Ae*. *aegypti* and *Ae*. *albopictus* from Brazil and Florida for an emergent Asian lineage of CHIKV. We carried out a series of experiments to determine two fundamental characteristics of this phenotypic trait: viral dissemination into the haemocoel of the tested mosquitos and saliva infection. These measurements characterize midgut and salivary gland barriers and are determinants of the vector competence of a mosquito population [26]. While viral dissemination indicates its propagation in the midgut and subsequent spread of the infection to other tissues, saliva infection is needed for the mosquito to successfully transmit the arbovirus by bite to a vertebrate host. Our results shed light on important questions regarding vector competence of *Aedes* mosquito populations of the Americas. The lack of statistical significance when comparing species and populations shows that viral dissemination occurs equally in these treatment conditions. In fact, more than 90% of all individuals have successful viral dissemination in their bodies, despite heterogeneity in species and population origin. This conclusion is further supported by the model results, which shows that none of the tested effects and interactions were statistically significant. Because high rates of disseminated infection were observed under these conditions, we had greater potential to detect treatment-dependent reductions in disseminated infection and less ability to identify treatment enhanced disseminated infection.

In our study, viral dissemination occurred rapidly, with around 85% of all individuals with positive legs at the 2nd day post-infection, and more than 98% of mosquitoes tested positive at the 13th day-post infection. Rapid viral dissemination together with a short extrinsic incubation period, as observed by saliva infection assays, may have important consequences for CHIKV epidemiology, especially given that both these *Aedes* species exhibit gonotrophic discordance [51, 52]. For instance, females will remain infectious for longer periods during the adult stage after ingesting CHIKV than pathogens with longer EIPs. Moreover, mosquito adult survival, EIP and host feeding strongly contribute to vectorial capacity which describes the number of infective bites received daily by a single host [53, 6]. A more thorough analysis showed that both populations of *Ae*. *aegypti* had similar levels of viral dissemination, reaching 100% of all tested individuals at the 5th day post-infection. For *Ae*. *albopictus*, we found a similar pattern with an increasing proportion of individuals with disseminated infection with each passing day post-infection. However, only the US population reached 100% of individuals with disseminated infection. This high number of individuals of both species and populations with disseminated infection might suggest a lack of substantial midgut escape barriers for the CHIKV strain used [31].

It is unclear whether differences in disseminated infection rates may be observed among these invasive *Aedes* mosquitoes if lower titer CHIKV infected blood were ingested. Studies have shown differences in susceptibility of *Aedes* vectors to CHIKV depending the dose of virus ingested [54, 55, 39]. Differences in susceptibility of *Ae*. *aegypti* and *Ae*. *albopictus* from Florida to infection and transmission of two lineages of CHIKV (Indian Ocean and Asian genotype) were tested [39]. In this study, *Ae*. *aegypti* tested with a lower dose of CHIKV Asian genotype in two different temperatures (25°C and 30°C) did not have significant differences in viral dissemination and transmission (100% to 40% and 33.3% to 0%, respectively). The low infection rates were attributed to a relatively low dose of CHIKV in blood meals (5.8 log_10_ pfue/ml). On the other hand, all populations of *Ae*. *aegypti* and *Ae*. *albopictus* presented higher susceptibility to infection and transmission for these two tested lineages of CHIKV at high titers [39, 54] determined the relative susceptibility of selected strains of *Ae*. *aegypti* and *Ae*. *albopictus* fed on a viremic monkey to infection with Southeast Asian strain of CHIKV. The results showed that strains of *Ae*. *albopictus*, regardless of their geographical origin, were more susceptible to infection (range, 72–97%) and dissemination (36–80%) with CHIKV than *Ae*. *aegypti* (infection rate, 12–25% and dissemination 8–25%) even though some strains presented lower infection rates in mosquitoes that ingested the lower dose (10^4.2–4.6^ pfu/ml). Coffey et al. (2014) [55] summarizes numerous chikungunya virus infection with *Ae*. *aegypti* and *Ae*. *albopictus*, stating lower and higher doses used in infected blood meals. In this review, the authors showed that infection, dissemination, and transmission rates of both *Aedes* vectors can vary according to the geographic sources of mosquitoes and the titer of the ingested bloodmeal. For instance, using bloodmeal titers of > 7 log_10_ pfu/ml (high dose) presented 80% of *Ae*. *aegypti* from all locations develop disseminated infection. For *Ae*. *albopictus*, more than half became infected or develop disseminated infection. The infection and dissemination rates for US *Ae*. *albopictus* are dose-dependent and seem to increase with the titer of the ingested bloodmeal [21, 40, 55]. Vega-Rúa et al. (2014) [31] assessed 35 American *Ae*. *aegypti* and *Ae*. *albopictus* for three CHIKV genotypes with the titer of 10^7.5^ pfu/ml, including mosquitoes populations from Brazil and Florida. Their study demonstrated that all 35 populations of both *Aedes* vectors were susceptible to CHIKV infection by all genotypes tested and that CHIKV transmission efficiency was highly heterogeneous in American mosquitoes ranging from 11.1% to 96.7%. Indeed, *Ae*. *albopictus* from Rio de Janeiro showed high transmission efficiencies even between geographically close populations, i.e., with some populations being able to transmit infectious viral particles as early as 2 days post-infection. However, the vector competence of *Ae*. *aegypti* and *Ae*. *albopictus* from Vero Beach was not tested for the Asian lineage of CHIKV, but for Indian Ocean and ancestral ECSA genotypes showed that transmission efficiencies were low (<30%).

The proportion of individuals with saliva infection was substantially lower than those with viral dissemination, suggesting salivary gland barrier(s) [31, 39]. Interestingly, US *Ae*. *aegypti* had almost twice as many infected individuals when comparing with the Brazilian population. A contrasting relation was observed for *Ae*. *albopictus*, with the Brazilian population reaching 60% of infected individuals against 24.5% from the US population. Thus, observed inherent differences in mosquito-virus interactions for both *Ae*. *aegypti* and *Ae*. *albopictus* might depend on geographic origin, which might impact disease transmission and contribute to its establishment in areas endemic for DENV and/or ZIKV. It is not clear whether heterogeneity exists in other traits that compose vector capacity, such as adult survival and biting rates, adult density, feeding behavior, and others, which would further influence CHIKV transmission and epidemiology in such areas [6]. Also, we observed that saliva infection declined with length of infection suggesting impaired transmission efficiency among older mosquitoes, most likely attributable to virus modulation of the infection as observed in other studies [56, 57]. Further studies on vector competence of *Ae*. *aegypti* and *Ae*. *albopictus* should be done to analyze the heterogeneity of dissemination and transmission of CHIKV among different populations of endemic or receptive areas for this arbovirus using a range of viral titers.

## Supporting information

S1 Fig Mean (SE) log 10 viral titer on disseminated CHIKV infection in *Ae*. *aegypti* and *Ae*. *albopictus* of Key West, Macapá, Okeechobee and Rio de Janeiro populations at 2, 5 and 13 days post-infection. (TIF)

S2 Fig Mean (SE) log 10 viral titer of CHIKV saliva infection in *Ae*. *aegypti* and *Ae*. *albopictus* of Key West, Macapá, Okeechobee and Rio de Janeiro populations at 2, 5 and 13 days post-infection. (TIF)
