# Supplementary figures and images for: Chikungunya virus vector competency of Brazilian and Florida mosquito vectors
Source: PLoS Negl Trop Dis. 2018 Jun 7;12(6):e0006521. doi: 10.1371/journal.pntd.0006521 (PMC6007930; doi:10.1371/journal.pntd.0006521)

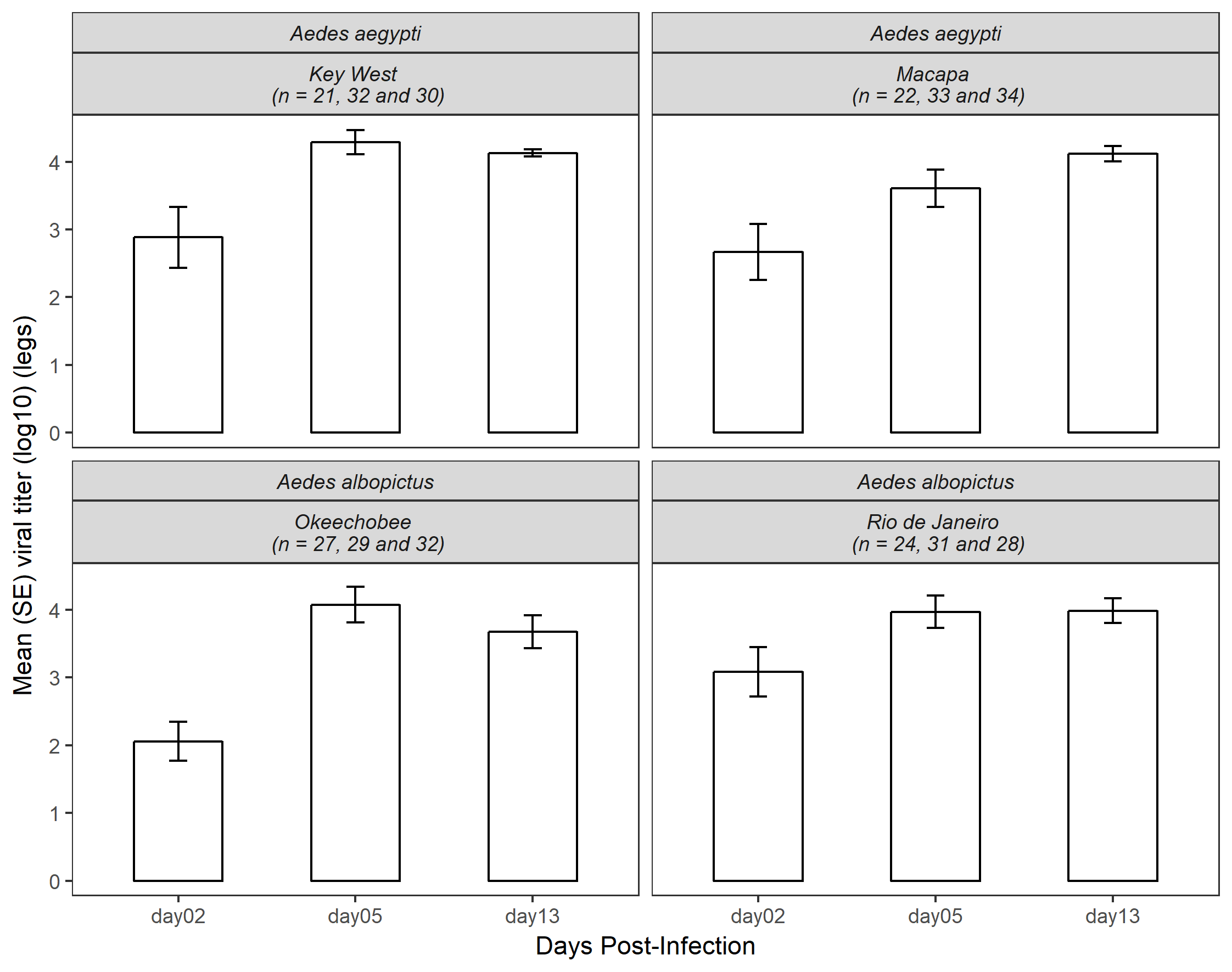

Supplement: S1 Fig — (TIF) [file pntd.0006521.s001.tif]

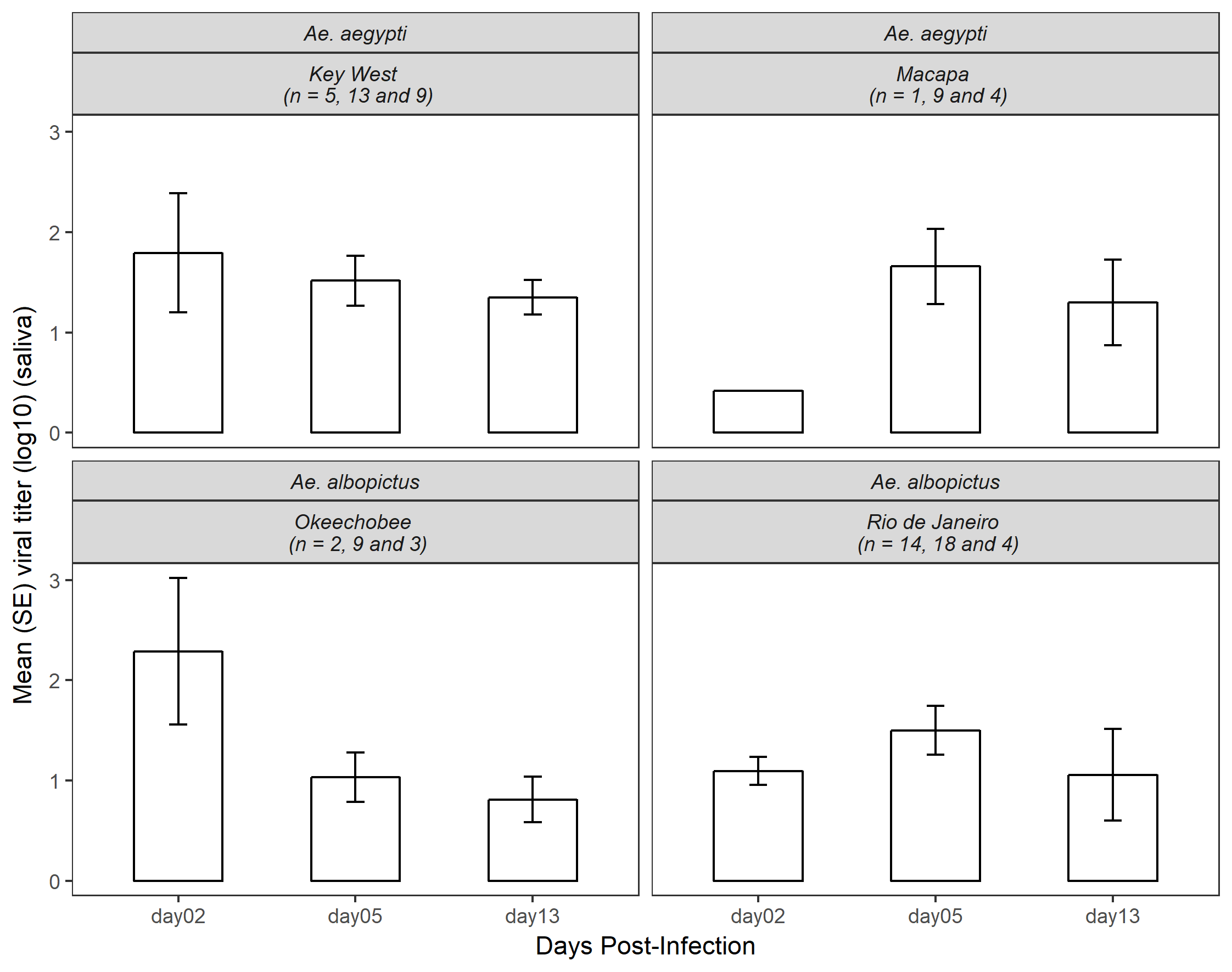

Supplement: S2 Fig — (TIF) [file pntd.0006521.s002.tif]
